# Organization of DNA in a bacterial nucleoid

**DOI:** 10.1186/s12866-016-0637-3

**Published:** 2016-02-20

**Authors:** Michael Y. Tolstorukov, Konstantin Virnik, Victor B. Zhurkin, Sankar Adhya

**Affiliations:** Department of Molecular Biology, Massachusetts General Hospital and Harvard Medical School, Boston, MA 02114 USA; Laboratory of Immunoregulation, Division of Viral Products, Office of Vaccines, Center for Biologics, FDA, Silver Spring, MD 20993 USA; Laboratory of Cell Biology, National Cancer Institute, National Institutes of Health, Bethesda, MD 20892 USA; Laboratory of Molecular Biology, National Cancer Institute, National Institutes of Health, Bethesda, MD 20892 USA

**Keywords:** Bacterial, Nucleoid, MNase, Digestion, Sequencing, Genomic, DNA, Packaging, Structural, Organization

## Abstract

**Background:**

It is unclear how DNA is packaged in a bacterial cell in the absence of nucleosomes. To investigate the initial level of DNA condensation in bacterial nucleoid we used in vivo DNA digestion coupled with high-throughput sequencing of the digestion-resistant fragments. To this end, we transformed *E. coli* cells with a plasmid expressing micrococcal nuclease. The nuclease expression was under the control of AraC repressor, which enabled us to perform an inducible digestion of bacterial nucleoid inside a living cell.

**Results:**

Analysis of the genomic localization of the digestion-resistant fragments revealed their non-random distribution. The patterns observed in the distribution of the sequenced fragments indicate the presence of short DNA segments protected from the enzyme digestion, possibly because of interaction with DNA-binding proteins. The average length of such digestion-resistant segments is about 50 bp and the characteristic repeat in their distribution is about 90 bp. The gene starts are depleted of the digestion-resistant fragments, suggesting that these genomic regions are more exposed than genomic sequences on average. Sequence analysis of the digestion-resistant segments showed that while the GC-content of such sequences is close to the genome-wide value, they are depleted of A-tracts as compared to the bulk genomic DNA or to the randomized sequence of the same nucleotide composition.

**Conclusions:**

Our results suggest that DNA is packaged in the bacterial nucleoid in a non-random way that facilitates interaction of the DNA binding factors with regulatory regions of the genome.

**Electronic supplementary material:**

The online version of this article (doi:10.1186/s12866-016-0637-3) contains supplementary material, which is available to authorized users.

## Background

Genomic DNA is highly compacted in both eukaryotic and prokaryotic cells. At the same time, genome needs to be accessible for properly controlled DNA metabolic processes, including DNA transcription, replication or recombination. DNA organization in eukaryotic chromatin has been extensively studied, resulting in the development of a hierarchical model of DNA compaction, starting from a nucleosome as a primary unit [1, 2]. In contrast, a bacterial analogue of the chromatin, the bacterial nucleoid, which comprises genomic DNA, RNA and associated proteins, has been considered in the past to be unstructured, with randomly folded DNA. However, the fact that genomic DNA is compacted 10^4^-times in the nucleoid [3] and, at the same time, it remains specifically accessible for interaction with regulatory factors suggests that the bacterial nucleoid is structurally organized in a non-random manner [4–13].

Generally, one can recognize several factors driving compaction of DNA in bacterial nucleoid: DNA supercoiling, macromolecular crowding, nucleoid-associated proteins, cellular polyamines, RNA, and specific sequence patterns in the genome affecting DNA flexibility [3, 14–20]. Moreover, the bacterial nucleoid exists in a dynamic state during cell growth, with the nucleoid volume in exponential phase being three-fold larger than in stationary phase [11]. Previous findings suggested that there are several levels of nucleoid compaction. Early studies of nucleoid organization at the macro-level employed electron microscopy to visualize DNA in the nucleoid, as well as nucleoid irradiation with sedimentation and trimethylpsoralen photobinding to monitor and measure supercoiling relaxation of the DNA [21–24]. They showed that 4.6-Mb circular *E. coli* genomic DNA in the nucleoid consists of 30-50 kb topologically independent supercoiled DNA domains at the highest level of compaction. Recent experiments using restriction endonucleases and recombination methods showed a lower level of nucleoid organization, represented by 10-kb topological domains, which are possibly branched and confined by dynamic barriers [25, 26]. Structures consisting of 40- and 80-nm fibers were visualized in nucleoids from cells in exponential and stationary phases by atomic force microscopy [11]. Other studies showed fine structure of DNA fibers and bigger loops that resembles nucleosome-like structure (“beads-on-string”) with 10-20-nm bead-particles corresponding to 200-300 bp condensed DNA blocks [27–30].

Advances in high-throughput technology, such as combining chromatin immunoprecipitation with either microarrays or next-generation sequencing (ChIP-chip and ChIP-Seq), provided powerful tools for genomics research including studies on bacterial nucleoid [31–35]. Several studies, employing ChIP-Seq and ChIP-chip methods, obtained extensive data on specific regions of *E. coli* genomic DNA bound by nucleoid-associated proteins such as H-NS, Fis, HU, IHF, etc [33, 34, 36, 37]. These studies revealed specific roles played by these proteins in establishment and maintenance of the nucleoid structure. However, the binding profiles reported in different studies varied considerably, probably due to differences in experimental conditions and complexity of the system, hindering an integrated analysis. In this study we employed a different strategy, focusing on the analysis of resistance of different nucleoid regions to enzymatic DNA digestions; such resistance is associated with the lack of access to the nucleoid fragments, allowing one to get information on the nucleoid structure.

Digestion assays are often used in the studies on nucleoid organization. In particular, micrococcal nuclease (MNase) has become an essential tool for probing DNA structure due to its relatively small size (about 17 kDa) compared to DNase I (about 30 kDa) and some other restriction enzymes [25, 35, 38–43]. Several studies that utilized digestion approach for the in vitro analysis of nucleoids extracted from such bacteria as *E. coli* and *B. subtitlis* demonstrated occurrence of DNA fragments of different sizes resistant to digestion [35, 44–49]. Sizes of the fragments ranged from high-molecular weight (possibly 10 kb) to about 100 bp, the latter being the most consistent result in the digestions. Similar results were obtained for other bacteria [45, 50], which suggest similar principles of genomic organization in different bacteria. However, these studies have not examined sequence composition and genomic location of the resistant fragments from the digested bacterial nucleoids.

**Fig. 1:**
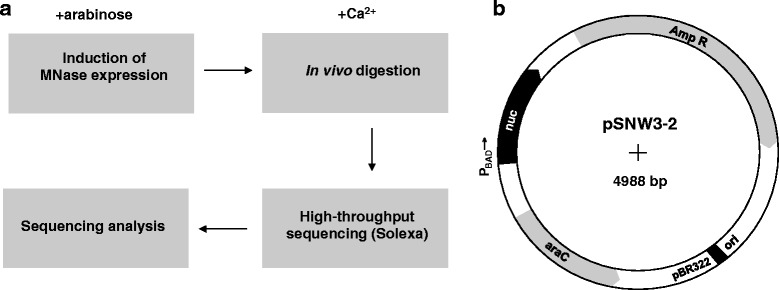
Study design. **a** Schematic representation of the major stages of the project. **b** Map of pSNW3-2 plasmid expressing MNase

Several structural models of bacterial nucleoid were suggested in the past [9–12, 25, 51–55]. However, these models were based on studies on the nucleoid organization at higher levels, not focusing on DNA packaging on the scales of hundred or thousand base pairs. In this paper we address the question of low-level structural organization of bacterial nucleoid. We developed a novel method of in vivo MNase digestion and applied it to the *E. coli* nucleoid coupled with high-throughput sequencing (Fig. 1a). We show that the DNA fragments protected from the digestion are non-randomly distributed in the *E. coli* genome and have sequence properties, which are distinct from bulk of the genome. Based on these results we propose a model of DNA organization in a bacterial cell where sequence-encoded structural properties of DNA play a key role.

## Results

### In vivo MNase digestion of *E. coli* nucleoids

Genomic DNA in *E. coli* nucleoid has sufficient accessibility to enzymatic cleavage in vivo as shown for restriction enzymes EcoRI and SwaI [25]. We selected MNase to test the *E. coli* genomic DNA organization in vivo because it was well-characterized in experiments on chromatin structure in eukaryotes [38, 42, 56]. MNase is a strictly dependent on Ca^2+^ endo-exonuclease that digests single-stranded and double-stranded nucleic acids preferentially at AT-rich regions, yielding 3’-nucleotides. Sequence preference of MNase is the same for naked DNA and for DNA in nucleosomes [57].

A map of the plasmid pSNW3-2 constructed for MNase expression is shown in Fig. 1b. pSNW3-2 is a derivative of pBAD24 plasmid used as a vector [58], into which MNase gene was inserted. MNase gene is expressed in this plasmid from the arabinose operon promoter *P*_*BAD*_, which is under the control of AraC repressor encoded in the same plasmid. Upon arabinose addition *P*_*BAD*_ is derepressed inducing MNase expression. Growth of the cells carrying pSNW3-2 in the presence of arabinose and Ca^2+^ was dramatically reduced because of high toxicity of MNase to the cells (Additional file 1: Figure S1a). Under conditions when the arabinose promoter is repressed, the growth was similar to that of wild type cells carrying an empty vector plasmid (Additional file 1: Figure S1b).

**Fig. 2:**
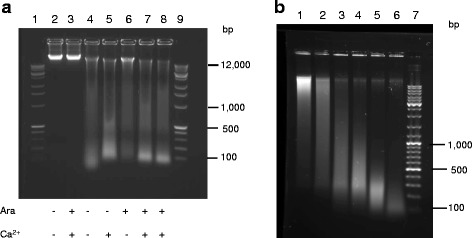
Analysis of the DNA fragments from in vivo MNase digestion of nucleoid in wild type *E. coli* (**a**) and in vitro MNase digestion of purified genomic DNA (**b**) in agarose gel. **a** Wild type cells with the empty vector (lanes 2, 3) and MNase-expressing vector (lanes 4-8) were supplemented with arabinose (lane 6), CaCl_2_ (lane 5) or both (lanes 3, 7, 8). Digestion reactions were stopped 1 minute (lane 7) or 5 minutes (lanes 3, 5, 8) after CaCl_2_ was added. Lanes 1 and 9 show DNA molecular weight marker. **b** Lane 1, purified wild type genomic DNA; lanes 2-6, a time course of in vitro MNase digestion of the wild type genomic DNA; lane 7, DNA molecular marker

After induction of MNase expression and nucleoid digestion, described in Methods, DNA digestion products were analyzed in agarose gel (Fig. 2a). Genomic DNA isolated from the cells containing only the vector showed no fragmentation whether arabinose and CaCl_2_ were added to the cells or not (Fig. 2a, lanes 2, 3). We observed a certain level of DNA fragmentation in the case when no CaCl_2_ or arabinose was added, likely due to leakiness of *P*_*BAD*_ in repressed state and presence of free Ca^2+^ in the LB-medium even with EGTA addition. Induction of the MNase expression in the cells with pSNW3-2 in the presence of Ca^2+^ allows extensive DNA fragmentation, giving DNA bands around 100 bp in size as measured by their electrophoretic mobility in agarose gel. DNA from this 100-bp band is resistant to the prolonged MNase digestion in vivo (Additional file 1: Figure S2). DNA from the 100-bp band was purified from the agarose gel and analyzed further. In vitro MNase digestion of genomic DNA purified from wild type cells (MG1655) was homogeneous with continuous fragmentation of longer DNA (Fig. 2b). To obtain a control DNA sample of similar size, purified genomic DNA was digested to an extent that majority of the DNA fragments form a 100-bp band in the agarose gel. This control was used for comparison to the experimental sample of in vivo digested DNA.

### Initial sequencing data analysis

**Fig. 3:**
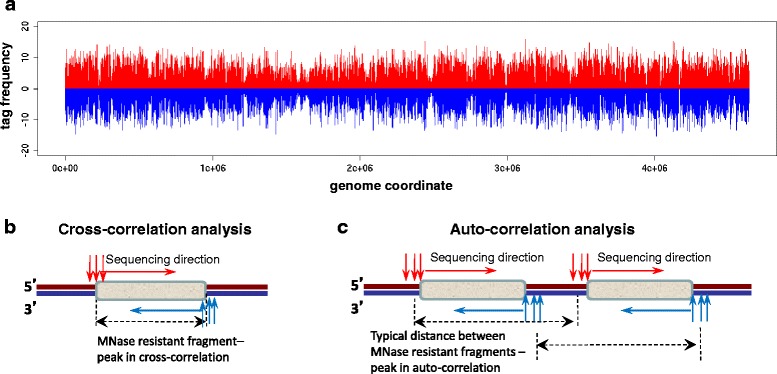
Genome-wide distribution of sequenced tags. **a** Tag frequencies for the entire *E. coli* genome. Frequencies of tags mapped on the positive and negative strands are shown with red and blue bars respectively. **a**, **c** Schematic illustrations of tag cross-correlation (**b**) and auto-correlation analyses (**c**). MNase resistant fragments are shown with grey rectangles. Vertical red and blue arrows represent 5’-ends of the digestion fragments mapping to the DNA positive and negative strands respectively

The 100-bp DNA fragments obtained after nucleoid digestion in vivo and purified genomic DNA digestion in vitro were subjected to massive parallel sequencing using Illumina/Solexa platform. The resulting sequence tags, which represent 36-bp-long 5’-ends of the digestion fragments, were aligned to the genome and only unique hits with no more than 2 mismatches were retained for the analysis. After removing potential artifacts in the sequenced tag distributions (see Methods for detail) the nucleoid and control samples comprised about 500 thousand and 3 million tags respectively. The tags cover the entire *E. coli* genome with the exception of repetitive DNA regions, which were excluded from consideration (Fig. 3a).

**Fig. 4:**
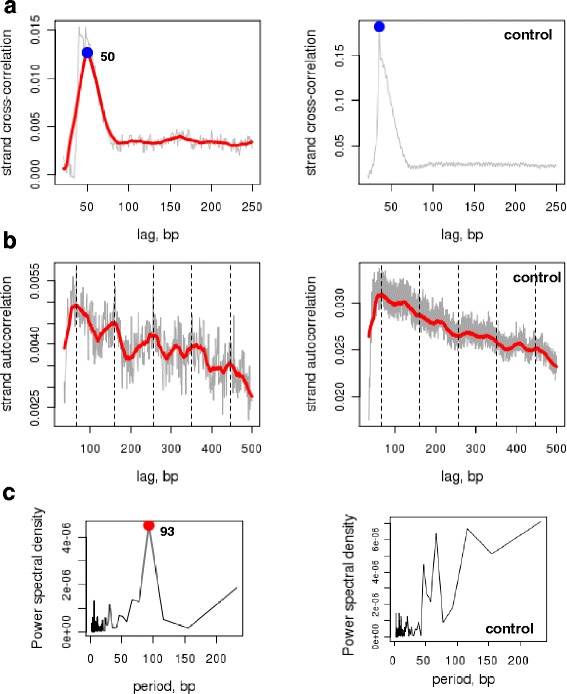
Correlation analyses for tags from the nucleoid and control samples. **a** Strand cross-correlations for the tags from the nucleoid sample and control. Grey line represents unsmoothed profile and red line corresponds to smoothing in 25-bp running window. The cross-correlation profile for the nucleoid has a non-trivial peak at 50 bp (the blue dot in the left panel). The tag cross-correlation profile for the control has only one peak corresponding to the tag length (the blue dot in the right panel). **b** Results of the strand auto-correlations analysis for the nucleoid sample and control. As in (**a**) grey line represents unsmoothed and red line represents smoothed profile. **c** Fourier analysis of the auto-correlation profiles shown in (**b**). The Fourier analysis reveals one dominant period of 93 bp in the auto-correlation profile for the nucleoid tags (indicated with the red dot)

To estimate the characteristic size of the MNase resistant fragments subjected to sequencing, we performed tag cross-correlation analysis [59]. This analysis identifies the most commonly-occurring distance separating a tag mapped to one DNA strand from the closest tag mapped to another DNA strand (Fig. 3b; distances are measured between digestion fragment centers). This analysis can be further explained as follows. The double-stranded digestion fragments included in the library can be sequenced from both ends resulting in two tags mapping to opposite DNA strands. Presence of such tag pairs can be detected by the cross-correlation analysis, allowing us to estimate the average distance between them and, thus, the characteristic length of the initial DNA fragments. The cross-correlation plots for both nucleoid and control samples feature a peak at 35-36 bp, which corresponds to the size of the sequenced tag (Fig. 4a). Such peaks are known to appear in cross-correlation analysis and likely originate from the requirement of unique tag alignment [60]. In addition to this ‘technical’ peak, there is a peak corresponding to the distance of 50 bp for the nucleoid sample (Fig. 4a, left panel). Remarkably, there is no ‘MNase-resistant’ peak in the control genomic DNA sample (Fig. 4a, right panel), suggesting absence of predominant fragment length and, thus, confirming specificity of the result described above for the nucleoid sample.

Next we performed the auto-correlation analysis for the sequenced tag distribution. While the cross-correlation analysis discussed above focuses on the tags mapped to different strands, the auto-correlation analysis focuses on the tags mapped to the same DNA strand (Fig. 3c) [59]. Specifically, this analysis estimates how often tags mapped to the same strand are separated by a certain distance and, thus, it allows revealing possible periodicities in the tag distribution. Our results show that there are distinct periodic peaks in the auto-correlation plot in the case of the nucleoid sample, while the plot for the control samples exhibits a nearly monotonic decline (Fig. 4b, right panel). We used Fourier analysis to quantitatively characterize the periodic signal detected by the auto-correlation analysis in the nucleoid sample. As it can be seen from the periodogram (Fig. 4c), which shows the relative contribution of the harmonics with different periods to the ‘aggregate’ oscillations, the main periodicity in the data corresponds to 93 bp (in other words, the digestion fragments frequently occur in groups where they are separated by the distances multiple to 93 bp). Again, as in the case of tag cross-correlation analysis, the control sample was used to confirm specificity of the observed periodicity for the nucleoid sample (Fig. 4c, right panel).

### Distribution of tags around 5’- and 3’-ends of genes

As discussed in the previous section, the sequence tags can be mapped to either positive or negative DNA strand. Since these tags are equivalent in regard to their representation of the actual digestion fragments produced by the MNase cutting of genomic DNA, it is useful to combine them for further analysis. To produce such a combined set of tags we computed the centers of the putative digestion fragments, adding half of the characteristic fragment size to the 5’-end position of each tag (see Methods for detail). The positions of the fragment centers do not bear any strand association and we use them in the analyses described below.

**Fig. 5:**
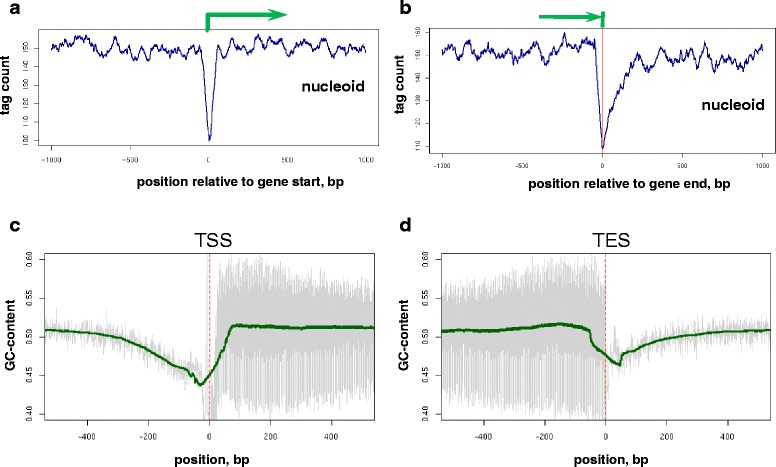
Distribution of the centers of the digestion fragments from the nucleoid sample relative to TSS (**a**) and TES (**b**). Positions of the fragment centers were estimated with the help of tag-cross correlation analysis (see Methods for details). Green arrows indicate direction of transcription. Positions of 5’-ends of the tags on the positive and negative strands were shifted by +/−25 bp respectively (the half-distance at which the maximum in cross-correlation profile is observed). The tag frequency profiles were smoothed by averaging in 50-bp running window. **c**, **d** GC-content of the MNase resistant fragments at TSS (**c**) and TES (**d**). GC-content is averaged for each position in the TSS and TES proximal regions over all annotated *E. coli* genes. Unsmoothed and smoothed profiles are shown with thin grey lines and thick green lines respectively

To investigate the distribution of location of MNase resistant fragments relative to the coding regions of the *E. coli* genome we determined the density of such fragments around transcription start and end sites (TSS and TES, respectively). The average profile for TSS-proximal regions revealed a sharp drop in the tag density centered at the transcription starts, which had the width of about 100 bp (Fig. 5a). The TES regions are also associated with a dip in tag density, which is asymmetrically wider in the downstream direction and has a somewhat smaller magnitude than the dip at TSS (Fig. 5b). Presence of these dips in the tag distributions is indicative of the increased accessibility of these regions for MNase and presumably other proteins.

These dips in fragment distribution could be artifacts of the MNase sequence preferences, since TSS and TES regions have sequence composition distinct from that of ‘bulk’ genomic DNA. To explore this possibility we compared the fragment density profiles at TSS and TES to the GC-content distribution and observed that the shape and exact locations of the dips do not coincide with the features of the GC-content profile (Fig. 5c, d). For instance, the dip in GC-content at gene starts is asymmetrically positioned relative to TSS unlike the symmetric dip in fragment density (Fig. 5a, c); it is also substantially wider than the fragment density dip. We conclude that the observed distribution of the fragment density around genes is specific, and while bearing certain degree of similarity to sequence composition profile, it cannot be completely explained by the latter.

### Clusters of enrichment of MNase resistant fragments

We next sought to identify all the regions where the MNase resistant fragments are enriched in the *E. coli* genome, without limiting our analysis by TSS and TES proximal regions. To this end, we estimated the enrichment ratios of the MNase fragment density in nucleoid sample over the values expected for randomized fragment distribution (see Methods for detail). Using the false discovery rate (FDR) threshold of 10^−3^, we identified 2087 regions where fragment density in nucleoid sample was significantly higher than that expected by chance and exceeded the tag density in the control sample (Additional file 1: Figure S3). The median size of the clusters was about 75 bp. Presence of such regions of enrichment in the genome can be interpreted as a tendency of the MNase resistant fragments to cluster together, rather than being randomly (or evenly) distributed in the genome.

**Fig. 6:**
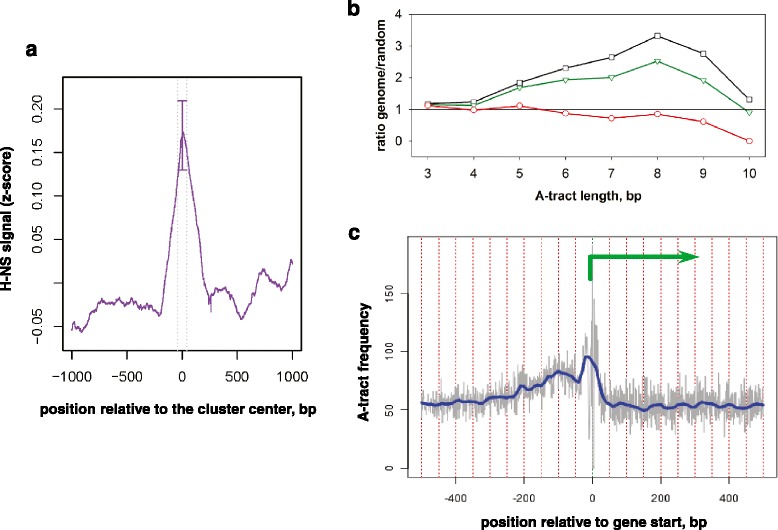
Properties of the clusters of enrichment in the MNase resistant fragments. **a** Average H-NS binding profile around the MNase resistant clusters. Analysis of association of the clusters with the H-NS binding was done using ChIP-seq data obtained in an independent study on the MG1655 *E. coli* strain in transition-to-stationary growth phase [33]. Y-axis represents relative signal of H-NS binding (z-score computed for the ChIP-Seq tag frequencies reported in [33]); x-axis represents distance from the cluster center. **b** Relative frequencies of A-tracts in the clusters of enrichment in MNase resistant fragments (red, circles), coding regions (green, triangles), and entire genome (black, squires). **c** A-tract frequency profile around gene starts (unsmoothed and loess-smoothed frequencies are shown with grey and blue lines, respectively). Green arrow indicates direction of transcription

To get insight into possible origins of MNase resistance of the genomic regions associated with the identified clusters we performed two types of analysis. First, we analyzed association of these regions with protein binding. We specifically focused on histone-like nucleoid-structuring protein (H-NS), for which the binding data was collected previously in a study with the experimental conditions similar to ours [33]. Our results show that H-NS binding is significantly enriched at the MNase resistant clusters (Fig. 6a), indicating a likely role of this protein in either formation or maintenance of DNA protecting nucleoid structures at these regions.

Second, we examined the sequence composition of the identified regions of fragment enrichment, focusing on such metrics as GC-content and A-tracts frequency in these regions. We note that the high GC-content correlates with increased DNA flexibility, and presence of A-tracts is associated with DNA curvature [61–65]. Our analysis showed that the GC-content of the clusters of enrichment was 51 %, which was close to the average for the *E. coli* genome. At the same time, these clusters were strongly depleted in short A-tracts as compared to entire genome and coding regions (Fig. 6b). Previously we have reported that the number of A-tracts calculated in for the entire *E. coli* genome is considerably higher than that expected from the nucleotide composition (the genome-to-random ratio, *r* > 3 for the A-tracts of 8-bp in length) [15]. The coding regions are also enriched for A-tracts (*r* > 1 for the A-tracts of most tested lengths); however, the genome-to-random ratio is lower for these regions than the genome-wide ratio. The analysis performed in this study revealed lower-than-expected frequency of A-tracts in the regions of enrichment in MNase resistant fragments (*r* ≤ 1), indicating that the genomic loci of high A-tract abundance were exposed to MNase digestion.

These observations led us to investigate the distribution of A-tracts relative to TSS of *E. coli* genes (see Methods). We observed the increase in A-tract frequency at gene starts with highest frequency at TSS and a wide increase in the promoter region (Fig. 6c). The presence of the peak in A-tract frequency at TSS co-localizes with the drop in the density of MNase-resistant fragments (Fig. 5a), which is consistent with our observation of the A-tract depletion within the regions of enrichment of resistant fragments. Taken together, our results demonstrate that the MNase resistant fragments are depleted in gene regulatory regions (Fig. 5a, b) and have specific sequence organization (Fig. 6b).

## Discussion

In this paper, we report that the DNA fragments protected from MNase digestion in *E. coli* nucleoid have non-random distribution and sequence organization. The characteristic size of such fragments is about 50 bp. The fragments tend to be clustered, rather than being evenly distributed in the genome. The most frequent distance between them is about 90 bp. We note that the periodicity in the digestion fragment distribution (i.e. tendency of the fragments to recur at regular intervals) and their clustering (i.e. tendency of the fragments to be located closer to each other than expected by chance) are distinct phenomena. Indeed, periodically located fragments may be evenly distributed along the genome, while clustered fragments may be distributed non-periodically. Since the density of the identified clusters is only 1 per about 2 kb and the median cluster size of about 75 bp (Additional file 1: Figure S3), it is unlikely that the periodically located fragments belong to different clusters. Observation of both periodicity and clustering in the tag distribution highlights its highly non-random organization.

**Fig. 7:**
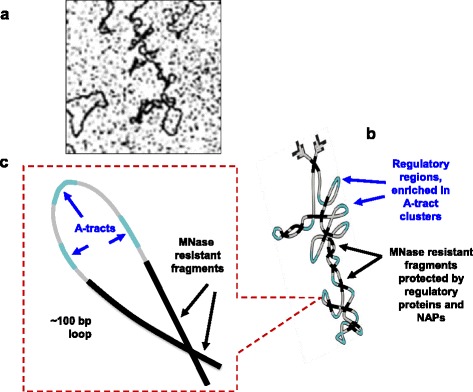
A possible model of low-level nucleoid organization. **a** A fragment of EM image of *E. coli* nucleoid (adapted from [21]). **b** Genomic DNA (gray) forms loops with A-tract clusters (cyan) located at apexes and MNase resistant fragments (black) occupying loop ‘stems’. **c** A blow-up of a DNA loop, containing several A-tracts (cyan) and two MNase resistant fragments (black)

The clusters of enrichment in MNase resistant fragments do not exhibit increased frequencies of A-tracts characteristic for the promoters of *E. coli* genes, and these fragments are depleted at gene starts and ends. At the same time, these clusters are associated with architectural protein binding (including H-NS binding sequences), which provides insight into possible molecular mechanisms responsible for increased DNA protection. Taken together, these data are consistent with a model, according to which MNase resistant fragments have limited accessibility due to steric protection by other DNA regions and/or interactions with other factors (proteins, RNA) and are preferentially absent in the gene regulatory regions (Fig. 7).

Remarkably the most frequent distance separating the MNase resistant fragments is close to 100 bp, the size detected for clusters of A-tracts in earlier analysis [15]. This suggests that the MNase resistant fragments may flank A-tract clusters, forming loop-like structures with easily accessible apexes (A-tract clusters) and flanks possibly involved in the interactions with ‘architectural’ proteins (Fig. 7). Such loops would be similar to those participating in gene expression regulation in some operons (e.g. *gal, lac, ara* loops etc.) and would represent the lowest level of structural organization of *E. coli* nucleoid [66–74].

## Conclusions

Unlike DNA organization in the eukaryotic chromatin, principles of compaction of bacterial genomic DNA in the nucleoid are still largely unknown. Perception of the bacterial nucleoid as an unstructured entity, consisting of randomly packaged DNA, RNA and associated proteins, has been changing lately toward a notion that the bacterial nucleoid is structurally organized. Existing models of bacterial DNA organization considered mostly high-level organization of the genomic DNA leaving low-level organization unclear. In this study we developed a novel method that utilizes in vivo MNase digestion combined with high-throughput DNA sequencing for the analysis of bacterial nucleoid organization at the lowest level. Using this approach we showed that the MNase resistant DNA fragments are non-randomly distributed in the *E. coli* genome. Specifically, the distribution of such fragments revealed a periodic character and a tendency towards clustering. These results allowed us to propose a model of hierarchical organization of the genomic DNA in the bacterial nucleoid. We suggest that the DNA loci preferentially protected against MNase digestion represent parts of the regulatory elements involving DNA loops like in *gal* and *gln*, which are occupied by “architectural” proteins like HU, IHF. These elements comprise 100-200 bp of DNA and are included in larger domains at the next level of nucleoid organization.

## Methods

### Bacterial strains and plasmid generation

DNA transformation with bacterial cells were performed in Max Efficiency DH5α competent cells (Invitrogen) except for the in vivo digestion experiments when wild type *E. coli* strain MG1655 was used. The gene encoding MNase was synthesized in PCR with a forward primer SNFR (5’-ccatactgctagcaggaggaattcaccatggcaacttcaactaaaaaattacataaag) and reverse primer SNRV (5’-gtcaagttctagattattgacctgaatcagcgttg). T4 phage DNA containing MNase gene (originated from pGN1526-1 plasmid) [75], a gift from Dr. Lindsay Black (School of Medicine, University of Maryland), was used as a template for the PCR reaction. The PCR product was cloned into plasmid pBAD24 between NcoI and XbaI restriction sites, giving plasmid pSNW3-2 containing MNase gene transcribed from the arabinose promoter. The cloned MNase gene contained three silent mutations, which did not significantly change codon frequency.

### In vivo MNase digestion

In our study of bacterial nucleoid, we employed DNA sequencing and subsequent analysis of DNA fragments obtained from in vivo MNase digestions of *E. coli* nucleoids. Briefly, wild type of *E. coli* was transformed with the MNase-expressing plasmid pSNW3-2 (a pBAD24 derivative) carrying MNase gene, which was expressed from the arabinose promoter *pBAD* under a tight regulation by AraC repressor encoded in the same plasmid [58].

Overnight culture of wild type *E. coli* with the MNAse-expressing plasmid was grown in LB-Lennox at 37 °C in the presence of 100 μg/ml ampicillin, 0.1 % glucose and 4 mM EGTA. The culture was diluted 1/50 and the cells were grown in LB-Lennox at 37 °C in the presence of 100 μg/ml ampicillin, glucose and 4 mM EGTA until the cell culture reached transition phase (OD about 1.7). MNase expression was induced by adding arabinose (0.1 % final concentration) to the growing cells for 30 min. Aliquots of 1.5 ml or 3 ml from the experimental culture and controls (MG1655 without the MNase-expressing plasmid and a culture grown without adding arabinose) were promptly collected, spun and washed in 1X PBS three times to remove LB and arabinose. Then cells were dissolved in 300 μl or 600 μl of PBS (for 1.5 ml and 3 ml of culture, respectively), ready for a digestion step.

The digestion reactions were initiated by adding 1 M CaCl_2_ to a final concentration of 2 mM and 100-μl aliquots were taken out during the time course of the digestion reactions. To quench the reactions 12 μl of 10 % solution of phenol in ethanol and EGTA to final concentration 25 mM were added on ice to each aliquot. Cells were harvested at 4 °C, treated with lysozyme and underwent several cycles of dry ice freezing-thawing to break the cells. RNAs and proteins were removed from the sample by intensive treatment first with RNase A and then with proteinase K. Resultant samples were run in 1.2 % agarose gel and the band containing the shortest DNA digestion fragments (around 100 bp) was cut out for subsequent DNA purification with QIAquick Gel Extraction Kit (Qiagen) and sequencing.

As control in sequence analysis, we used DNA fragments obtained from in vitro MNase digestion of purified wild type genomic DNA. The wild type genomic DNA was purified using Wizard Genomic DNA Purification Kit (Promega) and treated with MNase (New England Biolabs) at 12 °C. The aliquots were taken out and reaction was stopped at different time points by adding EDTA and incubating with proteinase K for 1 hour at 65 °C. In vitro digestion products were visualized in the agarose gel. DNA fragments with sizes comparable to those from in vivo digestion were purified directly from the reaction with QIAquick PCR Purification Kit (Qiagen).

### Sequencing and data analysis

The DNA fragments were sequenced using the Illumina/Solexa technology [76] (Genome Analyzer II) by PCPGM High Throughput Sequencing Facility (Cambridge, MA). The obtained short reads were mapped to the wild type *E. coli* genome allowing for two mismatches and only unique hits were retained. Positions in genome with the numbers of mapped tags above the significance threshold defined by a *Z*-score of 7 were identified as anomalous, potentially resulting from amplification bias, and the tags associated with them were discarded. The characteristic size of MNase resistant DNA fragments was estimated using the strand cross-correlation analysis and the periodicity in tag distribution was estimated with the strand auto-correlation analysis [59, 77].

The regions of significant tag enrichment in the nucleoid sample over the randomization were estimated as follows. Since the positions of sequenced tags correspond to 5’-ends of the DNA fragments submitted to sequencing, these positions were shifted by the half of the characteristic fragment size (50 bp) towards the fragment 3’-ends to represent centers of the DNA fragments. The positions from positive and negative DNA strands were combined. Then, tag density was calculated as a sum of Gaussian contributions that fitted tag frequencies at each genomic position. The bandwidth of 25 bp was used in the fit. The continuous regions of enrichment (clusters) longer than 25 bp were estimated using an FDR threshold of 10^−3^. Clusters separated by less than 25 bp were merged. To further validate these clusters, we identified and filtered out the clusters showing low enrichment of the fragment densities measured in the nucleoid sample versus the control sample. The final set comprised 2087 clusters. The distribution of the enrichment values (ratio of the tag densities in the nucleoid and control samples) is shown in Additional file 1: Figure S3b.

A-tracts were identified as sequences A_*n*_T_*m*_, where 3 bp ≤ (*n + m*) ≤ 10 bp. The genome-to-random ratio was estimated based on 15 implementations of the randomized ‘genomes’ of the same base composition as *E. coli* genome (see [15]).

## Additional file

Additional file 1: Figure S1. Overnight growth of wild type *E. coli* cells carrying empty or MNase-expressing vector on LB-agar-ampicillin plates at different conditions. **Figure S2.** A prolonged in vivo MNase digestion of nucleoid in wild type *E. coli* cells. **Figure S3.** The clusters of enrichment in the MNase resistant fragments. (PDF 1116 kb)

## Abbreviations

FDR: false discovery rate
MNase: micrococcal nuclease
TES: transcription end sites
TSS: transcription start sites
