# Supplementary material for: Organization of DNA in a bacterial nucleoid
Source: BMC Microbiol. 2016 Feb 20;16:22. doi: 10.1186/s12866-016-0637-3 (PMC4761138; doi:10.1186/s12866-016-0637-3)
Supplement: Additional file 1: Figure S1. — Overnight growth of wild type E. coli cells carrying empty or MNase-expressing vector on LB-agar-ampicillin plates at different conditions. Figure S2. A prolonged in vivo MNase digestion of nucleoid in wild type E. coli cells. Figure S3. The clusters of enrichment in the MNase resistant fragments. (PDF 1116 kb) [file 12866_2016_637_MOESM1_ESM.pdf]

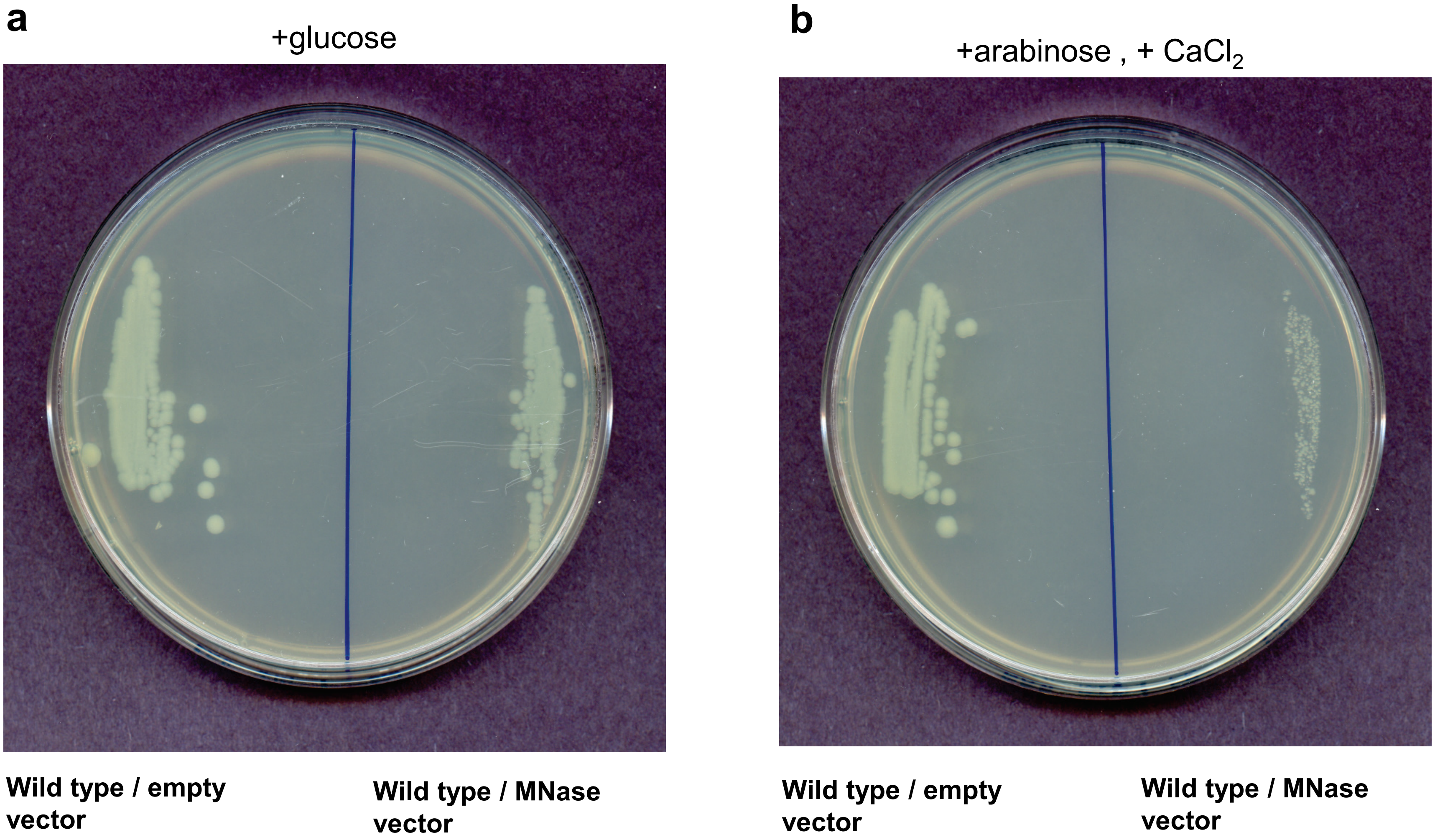

Supplementary Figure S1. Overnight growth of wild type *E. coli* cells carrying empty or MNase-expressing vector on LB-agar-ampicillin plates at different conditions. To suppress or induce MNase gene expression, the plates were correspondingly supplemented with glucose (**a**) or arabinose and $CaCl_2$ (**b**).

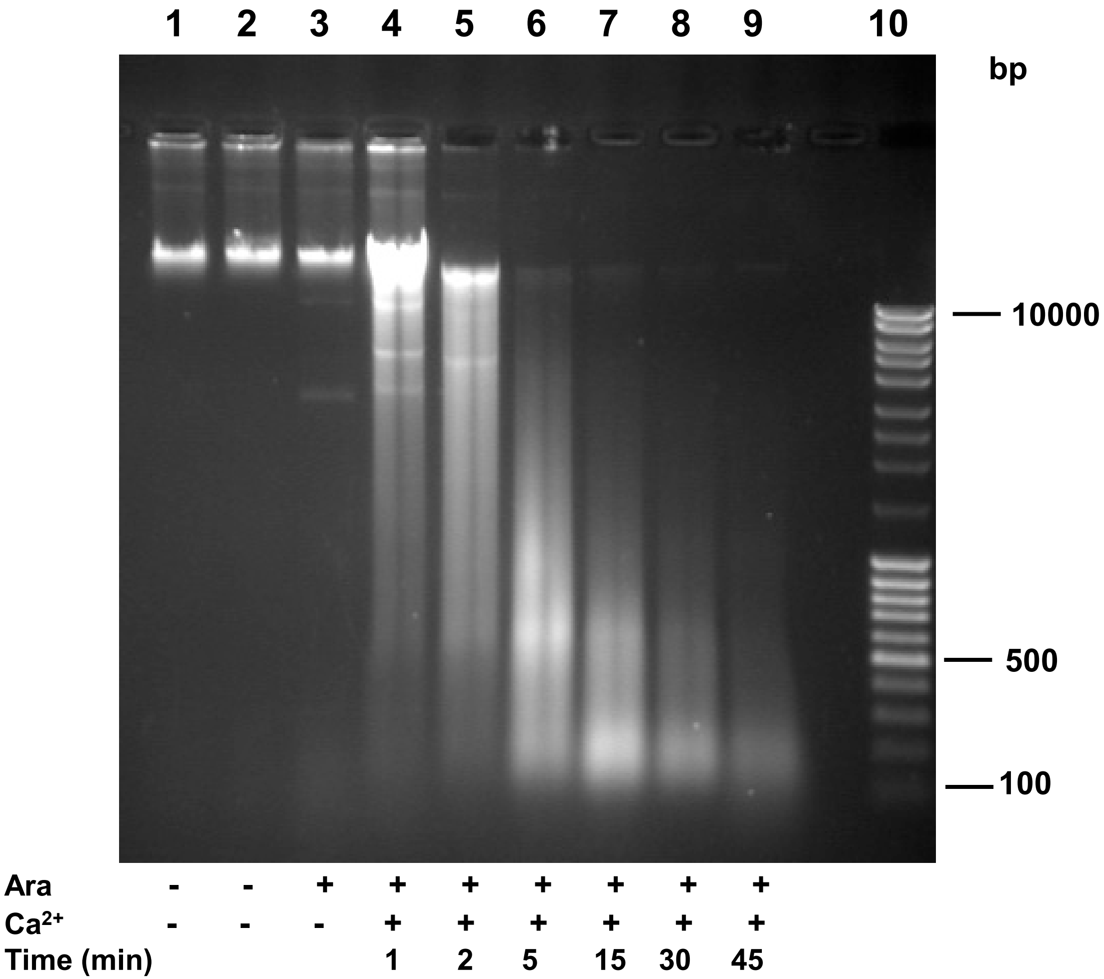

Supplementary Figure S2. A prolonged *in vivo* MNase digestion of nucleoid in wild type *E. coli* cells. Wild type cells with MNase-expressing vector (lanes 3-9) were supplemented with arabinose to induce MNase expression. $CaCl_2$ was then added to initiate digestion in all MNase-expressing samples but one (lanes 4-9). Digestion reactions were stopped 1, 2, 5, 15, 30 and 45 minutes later (lanes 4-9, respectively). DNA from wild type *E. coli* and DH5alpha *E. coli* with an empty vector are shown for comparison (lanes 1, 2). Lane 10 shows DNA molecular weight marker.

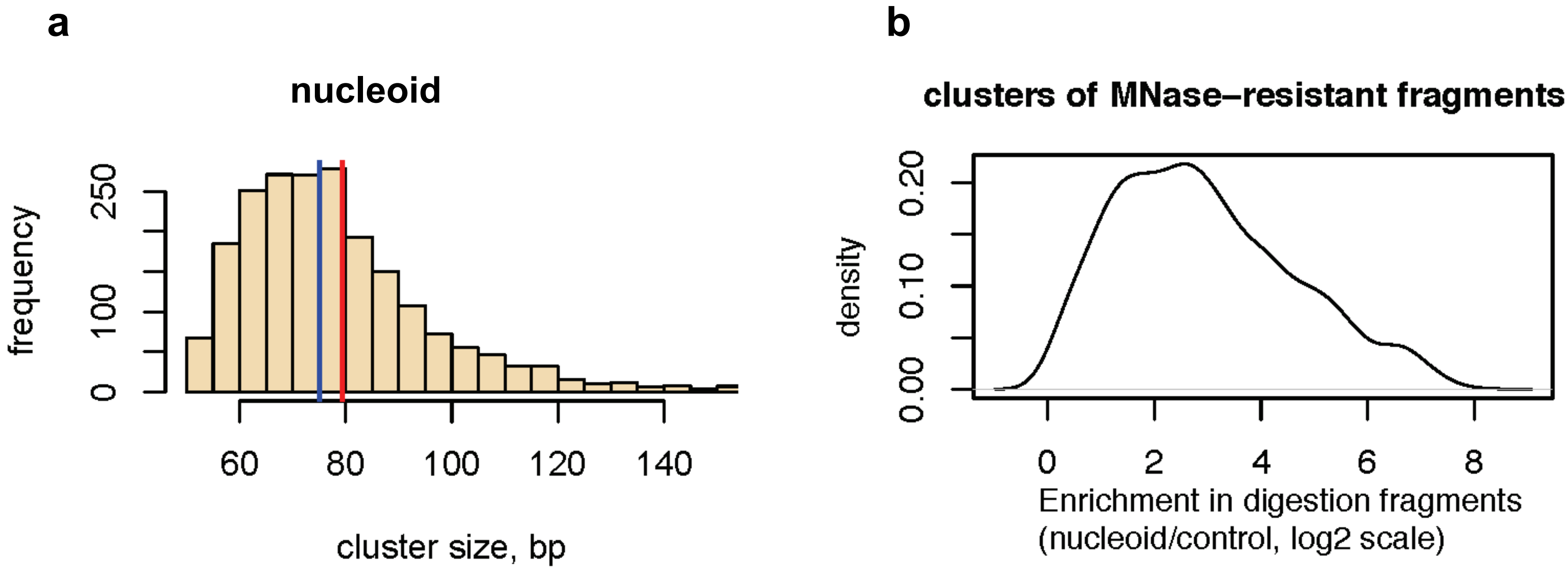

Supplementary Figure S3. The clusters of enrichment in the MNase resistant fragments. **(a)** Size distribution computed for the clusters of enrichment of MNase resistant fragments in *E. coli* nucleoid. Red and blue lines indicate mean and median sizes respectively. **(b)** The distribution of the enrichment values (ratio of the tag densities in the nucleoid and control samples).
